# Multi-Stage Tuberculosis Subunit Vaccine Candidate LT69 Provides High Protection against *Mycobacterium tuberculosis* Infection in Mice

**DOI:** 10.1371/journal.pone.0130641

**Published:** 2015-06-22

**Authors:** Hongxia Niu, Jinxiu Peng, Chunxiang Bai, Xun Liu, Lina Hu, Yanping Luo, Bingxiang Wang, Ying Zhang, Jianzhu Chen, Hongjuan Yu, Qiaoyang Xian, Bingdong Zhu

**Affiliations:** 1 Gansu Provincial Key Laboratory of Evidence Based Medicine and Clinical Translation & Lanzhou Center for Tuberculosis Research, School of Basic Medical Sciences, Lanzhou University, Lanzhou, China; 2 Institute of Pathogen Biology, School of Basic Medical Sciences, Lanzhou University, Lanzhou, China; 3 Lanzhou Institute of Biological Products, Lanzhou, China; 4 Department of Molecular Microbiology and Immunology, Bloomberg School of Public Health, Johns Hopkins University，Baltimore, Maryland, United States of America; 5 Koch Institute for Integrative Cancer Research and Department of Biology, Massachusetts Institute of Technology, Cambridge, Massachusetts, United States of America; 6 ABSL-3 Lab, Wuhan University, Wuhan, China; Public Health England, UNITED KINGDOM

## Abstract

Effective tuberculosis (TB) vaccine should target tubercle bacilli with various metabolic states and confer long-term protective immunity. In this study, we constructed a novel multi-stage TB subunit vaccine based on fusion protein ESAT6-Ag85B-MPT64(190-198)-Mtb8.4-HspX (LT69 for short) which combined early expressed antigens and latency-associated antigen. The fusion protein was mixed with an adjuvant being composed of N, N’-dimethyl-N, N’-dioctadecylammonium bromide (DDA) and polyriboinosinic polyribocytidylic acid (PolyI:C) to construct subunit vaccine, whose immunogenicity and protective ability were evaluated in C57BL/6 mice. The results showed that LT69 had strong immunogenicity and high protective effect against *Mycobacterium tuberculosis* (*M*. *tuberculosis*) H37Rv aerosol challenge. Low-dose (2 μg) of LT69 generated long-term immune memory responses and provided effective protection, which was even higher than traditional vaccine BCG did at 30 weeks post the last vaccination. In conclusion, multistage subunit vaccine LT69 showed high and long-term protection against *M*. *tuberculosis* infection in mice, whose effect could be enhanced by using a relative low dosage of antigen.

## Introduction

Tuberculosis (TB) remains a major global health problem and ranks as the second leading cause of death from an infectious disease [1]. Vaccination is still the most cost-effective approach towards improving public health in both industrialized and developing countries [2]. *Mycobacterium bovis* Bacillus Calmette-Gueerin (BCG) is the only TB vaccine for human use in current, but its protective efficacy wanes significantly over a period of 10–15 years [3, 4]. BCG vaccination mainly induces effector memory T cells (T_EM_) that survive for a shorter period of time than central memory T cells (T_CM_) [5], which may underlie the limited duration of BCG vaccine protection [6]. Therefore, novel vaccines and vaccine strategy should aim at inducing long-lasting T cell responses [7].

Subunit vaccines have been developed with the hope of boosting BCG-derived immunity so as to provide a long period of protection [8]. These vaccines include viral vector-based vaccines [9, 10] and recombinant proteins delivered with adjuvants. It was reported that the adjuvanted recombinant protein induced more memory/multifunctional T cells that lead to long-term immune memory responses compared to the viral vector in mouse and non-human primate TB models [11]. Therefore, recombinant protein-based subunit vaccine is perhaps the promising vaccine to provide long-term protection against TB.

T cell mediated immune response is believed to play an important role against *M*. *tuberculosis* infection. In response to vaccination, the majority of the activated T cells differentiate into antigen specific short-lived effector cells, whereas only a small proportion differentiates into long-lived memory cells [12, 13]. Antigen stimulation is the primary factor to regulate the diverse pattern of memory T cells [14]. Low-dose antigen and short-term antigen persistence were reported mainly to induce central memory T cell formation and hence facilitate development of long-term immunity *in vivo* [14, 15].

Our previous work showed that the combination of EAMM, which consists of four antigens highly expressed in replicating bacilli, and MH, which consists of dormancy-related antigen HspX, provided higher protective efficacy than EAMM or MH alone obviously [16]. These results suggest that vaccines combining antigens from both proliferation and dormant stages could generate broader immune responses and therefore could be more effective in eradicating all stages of *M*. *tuberculosis*. In this study, we fused EAMM and HspX together to construct a new multistage protein ESAT6-Ag85B-MPT64 (190–198)-Mtb8.4-HspX with molecular weight of 69kD, which was named as LT69. The immunogenicity and protective efficiency of it were evaluated in mice model.

## Materials and Methods

### Ethics statement

Animal experiments were performed in accordance with the guidelines of Council on Animal Care and Use. The protocols were approved by the Institutional Animal Care and Use Committee of Gansu University of Traditional Chinese Medicine (permit number: SYXK(Gan) 2013–001). Animals received free access to water and food throughout the study. During the experiments, the vaccinated and infected mice were monitored every day. Mice were euthanized by cervical dislocation.

### Bacterial strains

BCG Denmark strain was provided by Lanzhou Institute of Biological Products. *M*. *tuberculosis* H37Rv strain (ATCC 93009) was prepared by ABSL-3 Lab at Wuhan University. BCG and *M*. *tuberculosis* H37Rv were grown in Middlebrook 7H9 supplemented with oleic acid albumin dextrose catalase (OADC) (10% v/v) and glycerinum (0.5% v/v). Bacterial suspensions were frozen and stored at -80 ℃. Serial dilutions of the bacteria suspensions were plated on 7H11 OADC agar plates for colony forming units (CFU) counting before use.

### Animals

C57BL/6 female mice (6–8 weeks old) were purchased from Slaccas Inc. (Beijing, China) and were maintained in special pathogen-free conditions in Gansu University of Traditional Chinese Medicine. For *M*. *tuberculosis* H37Rv challenge experiments, animals were kept in ABSL-3 lab at Wuhan University.

### pET30a(+)-ESAT6-Ag85B-MPT64(190–198)-Mtb8.4-HspX plasmid construction

Recombinant pET30a(+)-Mtb8.4-HspX and pET30a(+)-ESAT6-Ag85B were produced as previously described [16]. The plasmid encoding ESAT6-Ag85B-MPT64(190–198)-Mtb8.4-HspX was generated by inserting the gene fragments into the multiple cloning sites of pET30a(+) successively as follows. Initially, the DNA sequences of MPT64(190–198)-Mtb8.4-HspX was generated by PCR amplification from pET30a(+)-Mtb8.4-HspX plasmid with the primer MMH F, 5’-3’GA***AGATCT*** TTCGCAGTCACGAACGACGGGGTGATTAGGCTGTCGTTGACCGCA（*Bgl* II）and the primer MMH R, 5’-3’TAGGCAAGCTTTCAGTTGGTG GACCGGAT （*Hin*d III), the sequence of MPT64190-198 is underlined. The fragment was cloned into the *Bgl* II and *Hin*d III site of pET30(+) to construct the plasmid pET30(+)-MPT64(190–198)-Mtb8.4-HspX. ESAT6-Ag85B sequence was generated by PCR amplification from pET30a(+)-ESAT6-Ag85B plasmid with the primer EA F, 5’-3’ CGGCATATGACAGAGCAGC AGTGGAA T (*Nde* I) and EA R, 5’-3’ GAAGATCTGCCGGCGCCTAACGA ACTCTGGAG(*Bgl* II). Then this fragment was cloned into the unique sites *Nde* I and *Bgl* II of the previously constructed pET30(+)-MPT64(190–198)-Mtb8.4-HspX plasmid to get the last plasmid. The final plasmid was transformed into the *E*. *coli* strain BL21 to express the fusion protein LT69.

### Expression and purification of mycobacterial fusion proteins

*E*. *coli* BL21 expressing LT69 was incubated with 0.5 mM isopropyl β-D-thiogalactopyanoside (IPTG) for 6 h at 25 ℃. Then, cells were harvested and sonicated. Finally, the supernatant containing the target protein LT69 was subjected to purification as below.

Two steps were involved in the purification of LT69. First, saturated ammonium sulfate was added to the protein sample to 8% of saturation, and followed by centrifugation. The supernatant was discarded and the pellet was resuspended in buffer A (phosphate buffer, 20 mM; pH7.4). The resuspended pellet was subjected to ammonium sulfate precipitation using 6%, 4% and 2% saturation successively. The final precipitate was collected and resuspended in buffer A containing 0.02 M arginine and 1 M urea. Second, the LT69 was further purified by a gel filtration chromatography (GFC) on Superdex 75 column. At last, the protein LT69 was eluted from the resin with buffer B (phosphate buffer, 20 mM; sodium chloride, 0.15 M; pH7.4). Endotoxin level of the fusion protein LT69 was quantified by Gel Clot Tachypleus Amebocyte lysate (TAL) assay (Zhanjiang A&C Biological Ltd., Zhanjiang, China). MH and EAMM were purified as described previously [16, 17].

### Immunogenicity and protective efficacy of the fusion protein LT69 in adjuvant of DDA-Poly (I:C)

Mice were injected with 10 μg of LT69 emulsified in an adjuvant being composed of N, N'-dimethyl-N, N'-dioctadecylammonium bromide (DDA) (250 μg/dose; Sigma-Aldrich, Poole, UK) and polyinosinic-polycytidylic acid [Poly (I:C)] (50 μg/dose; Sigma-Aldrich, Poole, UK) subcutaneously with a total volume of 200 μl/mice. Mice received 10 μg of EAMM plus 10 μg of MH, BCG (5 × 10^6^ CFU) and PBS were served as controls. BCG was injected once at week 0. Other groups received three times inoculations at week 0, 2, and 4 respectively.

Six weeks after the last immunization, the levels of antigen-specific IFN-γ, IL-2 and antibodies were analyzed. IFN-γ was tested using an enzyme-linked immunospot (ELISPOT) assay following 20-h incubation of splenocytes with special antigens including ESAT-6 (10 μg/ml), Ag85B (5 μg/ml), Mtb8.4 (10 μg/ml), HspX6 (10 μg/ml) and PPD (5 μg/ml). IL-2 secretion was detected by ELISA assay from the spleen cells culture supernatant. The levels of IgG1 and IgG2c antibodies against ESAT6 (10 μg/ml), Ag85B (5 μg/ml) and HspX (10 μg/ml) in mouse sera were determined using ELISA assay as described previously [17].

Thirty weeks after the last immunization, mice were challenged with virulent *M*. *tuberculosis* H37Rv *via* the respiratory route at approximately 50–100 CFU per mouse. For infection, frozen bacteria stocks were thawed and diluted to 10^6^ CFU/mL, and nebulized in an aerosol infection chamber (Salter Labs，CA, USA) containing the mice. Infectious dose was determined by plating whole lung homogenates on day 1. Ten weeks later, the animals were euthanized, and lungs and spleens were homogenised in saline and plated at 10-fold serial dilutions on Middlebrook 7H11 agar (BD, NJ, USA) enriched with OADC (BD, NJ, USA) and an antibiotic mixture including carbenicillin, trimethoprim and amphotericin B (Sigma, MO, USA).

### Immune memory responses induced by different doses of LT69

LT69 vaccine was used for vaccination in doses of 2 μg, 10 μg and 50 μg in 200μl separately. Mice were vaccinated subcutaneously three times with 2 weeks apart. Control mice received PBS or a single dose of 5 × 10^6^ CFU BCG. Six weeks and twenty-four weeks after the last injection, the antigen specific IFN-γ secretion was detected by ELISPOT as described above.

### Statistical analysis

The results were expressed as means ± SD. Data were compared using analysis of variance (ANOVA) and SPSS13.0 software. Values of *p* < 0.05 were considered as statistically significant.

## Results

### Expression and purification of LT69, EAMM and MH

**Fig 1:**
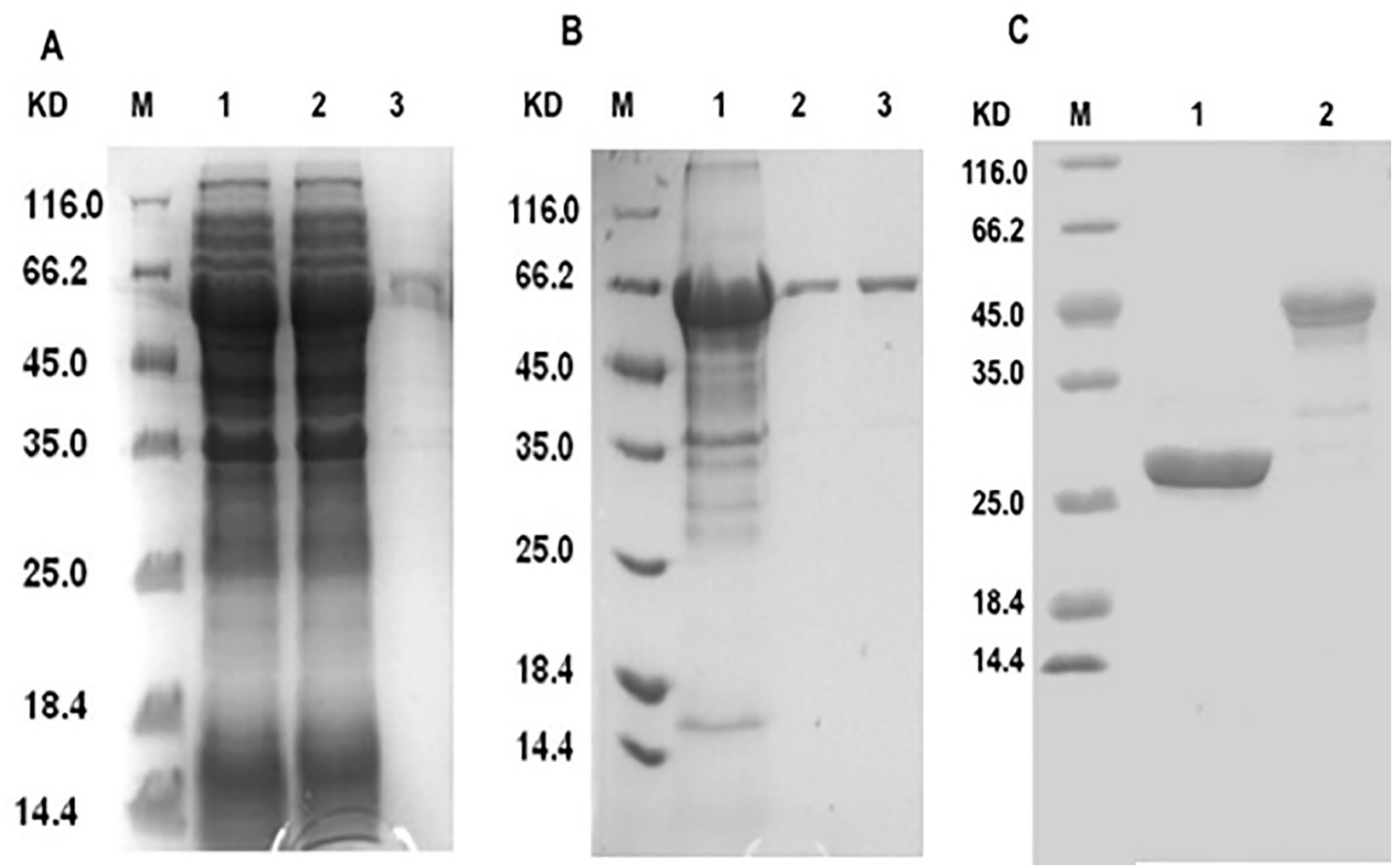
Expression and purification of LT69. (A) Expression of LT69 in *E*. *coli*. The LT69 protein was induced by IPTG in *E*. *coli* BL21 at 25℃ for 6h. Coomassie Blue-stained 12% SDS-PAGE of total *E*. *coli* lysate (lane 1), supernatant of *E*. *coli* lysate (lane 2), and sediment of *E*. *coli* lysate (lane 3). (B) Purification of LT69. LT69 was purified by two steps, and finally its purity is beyond 90%. The figure shows a Coomassie Blue-stained 12% SDS-PAGE of LT69 purified by gradient salt fractionation (*lane* 1) along with LT69 purified by gradient salt fractionation and gel filtration chromatography (*lane* 2, 3). M, molecular weight. (C) Purified fusion proteins MH (*lane* 2) and EAMM (*lane* 3).

The fusion protein LT69 could be expressed in *E*. *coli* in supernatant (Fig 1A) and was purified by gradient salt fractionation and gel filtration chromatography successively (Fig 1B). The fusion proteins EAMM and MH [17] were purified and confirmed by SDS-PAGE (Fig 1C) and Western-blot (data not shown). At a concentration of 0.4 mg/mL LT69, the endotoxin level was less than 2.5 EU/ml, which was lower than the limit for animal and in *vitro* experiment required.

### LT69 was of strong immunogenicity and high protective efficacy

**Fig 2:**
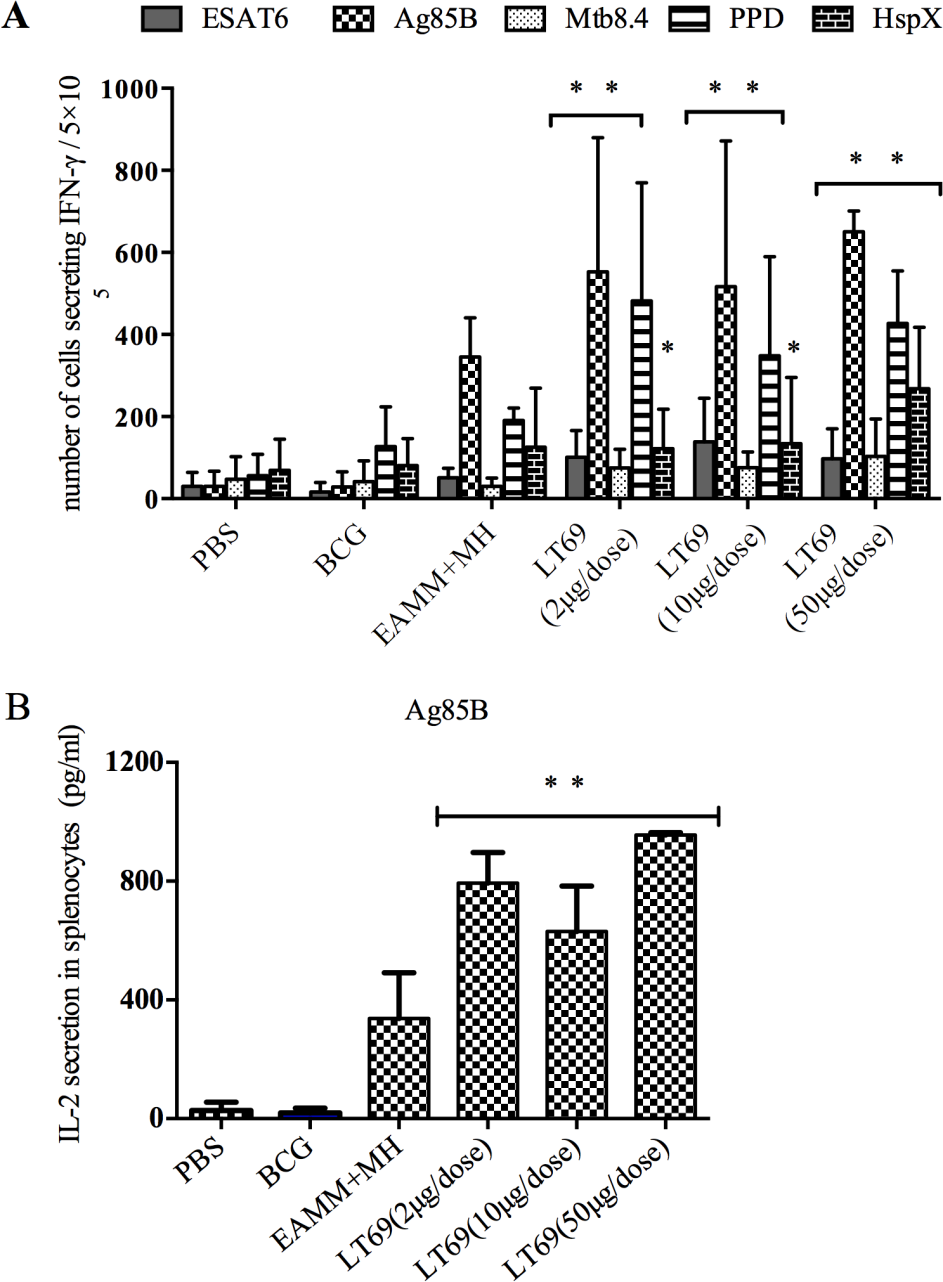
The immunogenicity of LT69 vaccine in mice. C57BL/6 mice were immunized with 50μg or 10μg or 2μg of LT69 formulated in DDA/Poly(I:C) separately via subcutaneous injection three times (2 weeks apart) or with a single dose of BCG (5×106 CFU). For EAMM+MH group, the mice received EAMM (10 μg) plus MH (10 μg) in DDA/Poly(I:C). Six weeks after the final immunization, spleen cells were stimulated with ESAT6 (10 xg /ml), Ag85B (5 μg/ml), Mtb10.4 (10 μg/ml), HspX (10 μg/ml) and PPD (5 μg/ml) separately *in vitro*. (A) The IFN-γ secretion in splenocytes. (B) The IL-2 secretion in splenocytes. Results are presented as means ± SD, n = 4. * *p* < 0.05, relative to PBS，BCG; ** *p* < 0.05, relative to PBS，BCG and EAMM+MH groups

With the stimulation of ESAT6, Ag85B, Mtb8.4 or PPD in *vitro*, the production of IFN-γ and IL-2 by spleen cells from mice immunized with LT69 were higher than that from PBS, BCG and EAMM+MH (*p* < 0.05) (Fig 2). With the stimulation of HspX, the IFN-γ secretions in the group of LT69 were higher than PBS, and BCG groups, but the differences between the groups of LT69 and EAMM+MH were not obvious (Fig 2A).

IgG1 and IgG2c antibody titers against antigens ESAT6, Ag85B and HspX and the relative ratio of IgG2c titer to IgG1 titer in LT69 group were significantly higher than that of BCG group (*p* < 0.05) and almost as same as EAMM+MH group (Table 1). The antibody titers in PBS control group were negative.

**Table 1:** Serological responses to antigens in LT69 vaccine immunized mice.

	Anti-ESAT6			Anti-Ag85B			Anti-HspX		
	IgG1	IgG2c	IgG2c/IgG1	IgG1	IgG2c	IgG2c/IgG1	IgG1	IgG2c	IgG2c/IgG1
PBS	-	-	-	-	-	-	-	-	-
BCG	2.00	< 2	-	< 2	< 2	-	< 2	< 2	-
EAMM+MH	4.25±0.71	4.33±0.79	1.01±0.03	3.66±0.52	2.00	0.56±0.09	3.88±0.57	0.56±0.09	0.62±0.1
LT69(2 μg) *	3.40± 1.14	4.51±0.62	1.4±0.38	3.96±0.52	3.43±0.15	0.88±0.14	4.18±0.15	0.88±0.14	0.75±0.04
LT69(10μg) *	4.48±0.67	4.41±0.60	0.95±0.07	4.10±0.25	3.13±0.62	0.77±0.19	4.56±0.52	0.77±0.19	0.67±0.04
LT69(50μg) *	4.55±0.3	4.31±0.69	0.91±0.15	4.41±0.25	3.12±0.62	0.71±0.17	4.41±0.81	0.71±0.17	0.63±0.2

*P < 0.05, relative to PBS and BCG groups

**Fig 3:**
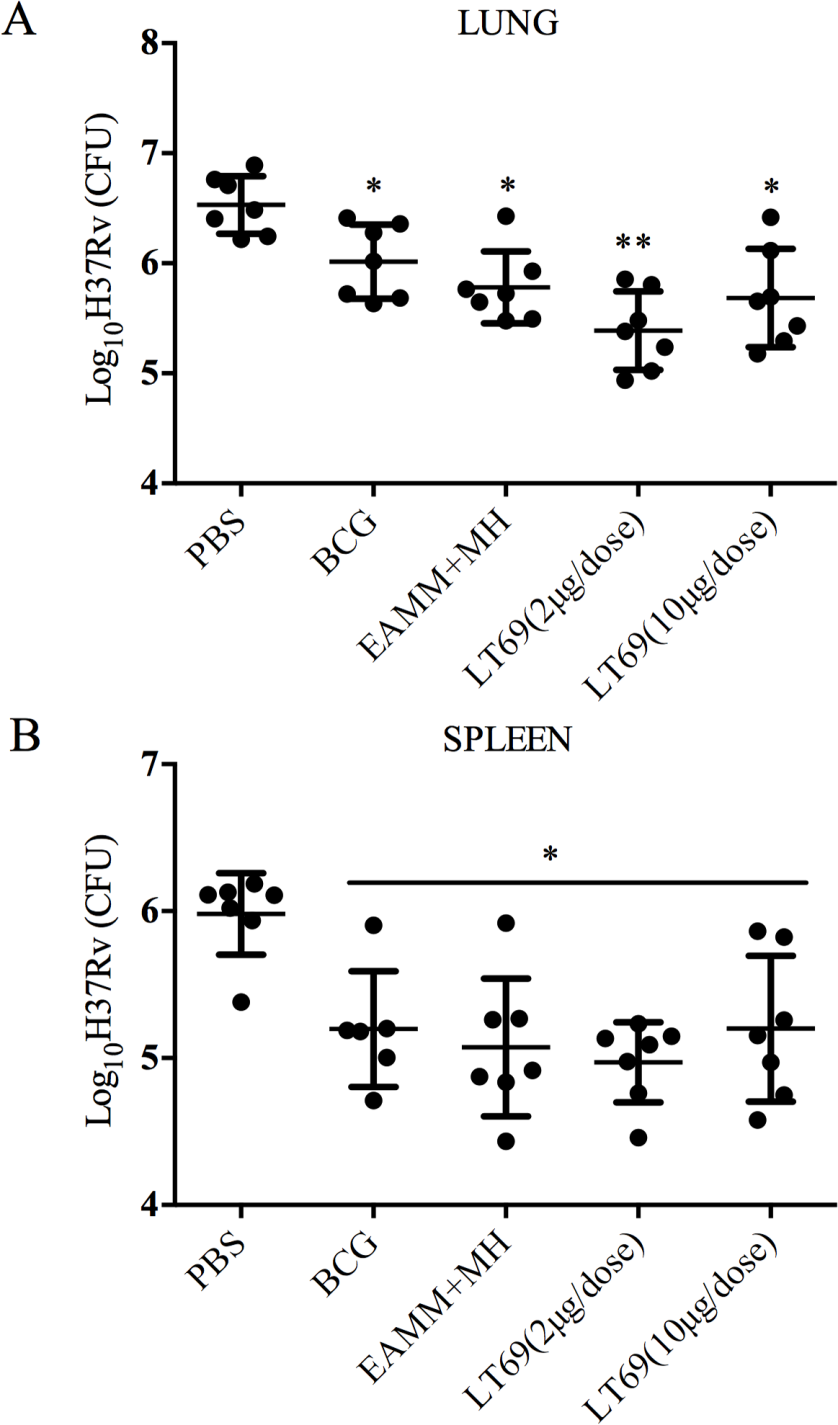
The protective efficacy of LT69 against *M*. *tuberculosis* H37Rv infection in mice. Mice were immunized subcutaneously with two different doses (10 μg and 2 μg) of LT69 formulated in DDA-Poly I:C three times at 2-wks interval or with a single dose of BCG (5×106 CFU). At the 34th week, 30 weeks after the last vaccination, mice were aerosol-infected with *M*. *tuberculosis* H37Rv 50–100 CFU. Ten weeks later, the protective efficacy was measured and was expressed as log10 of number of CFU in lungs (A) and spleens (B). Results are presented as means ± SD from groups of seven mice. * *p* < 0.05, relative to PBS; ** *p* < 0.05, relative to PBS，BCG and EAMM+MH groups.

Furthermore, 30 weeks after the last vaccination, we detected the protective effect of LT69 vaccine using the *M*. *tuberculosis* H37Rv aerosol challenge model. Subunit vaccine LT0069 in dose of 2 μg showed higher effective rate of protection against *M*. *tuberculosis* than EAMM+MH and BCG did (*p* < 0.05), with 1.17 log_10_ CFU decline in the lungs compared with PBS control, while LT69 in 10 μg per dose showed the same protective efficacy as EAMM +MH and BCG, reducing approximately 0.85 log_10_ CFU of the bacilli in lungs compared with PBS control (Fig 3A).

### Low-dose LT69 generated long-lived cellular immune responses

**Fig 4:**
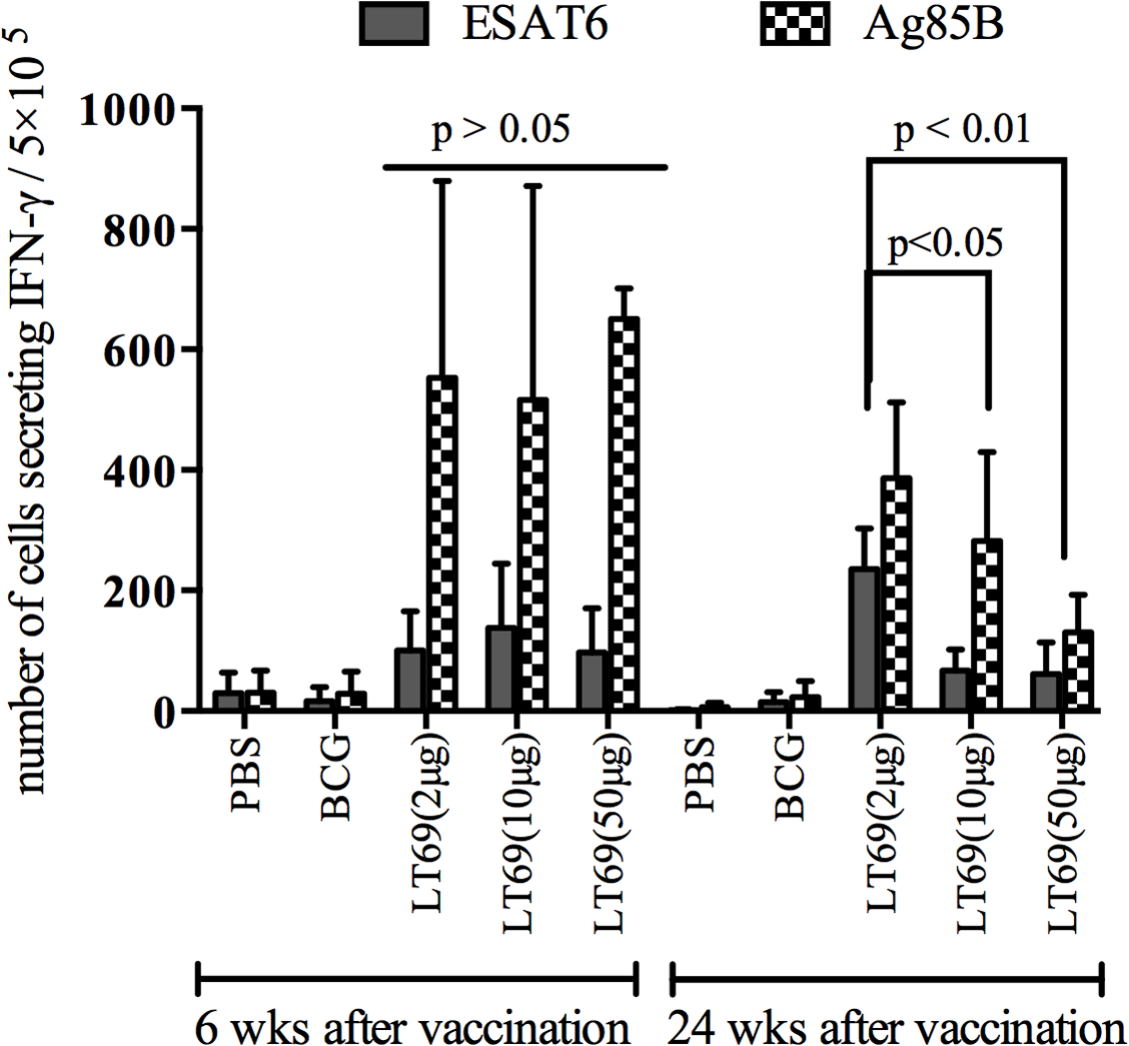
The T cell immune responses in mice immunized with different dosage of LT69. Mice were immunized subcutaneously with three different doses（50 μg, 10 μg or 2 μg) of LT69 formulated in DDA-Poly I:C three times at 2-wks interval or with a single dose of BCG (5×106 CFU), and the number of vaccine-induced IFN-γ cells was determined at 6 and 24 weeks after the last immunization. Freshly isolated spleen lymphocytes were plated in duplicate at 5×10^5^ cell per well in 96 spot and incubated with ESAT6 (10 μg/ml), Ag85B (5 μg/ml) and HspX (10 μg/ml) for 20h. IFN-γ production was determined using mouse IFN-γ ELISPOT kits. Results are presented as means ± SD, n = 4

To examine the T cell immune memory induced by different dosages of LT69, mice were vaccinated with three doses of LT69 (2 μg, 10 μg or 50 μg) separately. Antigen specific cellular immune responses were detected twice at 6 and 24 weeks after the last immunization, respectively. Six weeks after the last inoculation, all the LT69 vaccinated mice had high levels of IFN-γ releasing from antigen stimulated splenocytes *in vitro* and there was no obvious difference among them (Fig 4). However, 24 weeks after the last immunization, the 2 μg of LT69 vaccinated mice generated significantly higher levels of IFN-γ than the mice received 10 μg and 50 μg of the vaccine (*p* < 0.01)， and the 10 μg group generated higher immune responses than that of the 50 μg group (*p* < 0.05) (Fig 4). As for protective efficacy, 2 μg of LT69 immunization reduced more bacteria load in lung tissues than BCG and EAMM+MH did, but 10 μg of LT69 did not show so strong protection (Fig 3A).

## Discussion

In this study we recombined our previous fusion protein EAMM and MH together to construct a novel multistage fusion protein LT69. We found that LT69 had at least the same levels of immunogenicity and protective efficacy against *M*. *tuberculosis* infection as EAMM plus MH did. Especially, low dosage of LT69 (2 μg/dose) induced higher long-term protective efficacy than traditional BCG at 30 weeks post vaccination.

According to the Yin-Yang model proposed, in either active TB or latent TB, the internal bacterial population consists of varying growing and non-growing sub-populations with different metabolic states and these populations can interconvert with each other [18]. Therefore, TB subunit vaccines should combine antigens in various growth stages of *M*. *tuberculosis* so as to provide protective immunity against bacteria in different metabolic states. In this study, LT69 vaccine consists of four antigens (ESAT6, Ag85B, Mtb8.4, and HspX) and a CD8 epitope (the 190–198 peptide of MPT64), which are the main immunodominant antigens expressed separately in replicating and dormant *M*. *tuberculosis*. Mice receiving LT69 generated robust antigens specific IFN-γ and IL-2 responses as well as high titers of antigen-specific antibodies, indicating that the novel vaccine consisting of five antigens had strong immunogenicity and the single antigens can be recognized well by LT69 induced immunity. Furthermore, LT69 had at least the same level of protective efficacy against *M*. *tuberculosis* infection as EAMM+MH and BCG vaccine（Fig 4A). All these suggested that LT69 vaccine might be a promising subunit vaccine to prevent TB by targeting several metabolic states of infected bacilli.

Besides generating broad and strong protective immunogenicity against infected bacilli with different metabolic states, multi-component vaccine LT69 might also be predicted having high population coverage. *M*. *tuberculosis* antigens could not be recognized by immune cells from all TB patients because of the polymorphism of human leukocyte antigen (HLA) system. Vaccine combining more than one antigen has the advantage of being recognized by extensive human populations. In addition, there is still the possibility that vaccine based solely on one antigen may select for *M*. *tuberculosis* that do not express the antigen so as to generate immune evasion. LT69 containing five *M*. *tuberculosis* antigens might be beneficial for vaccination of genetically diverse human populations and overcoming antigen presentation evasion [19, 20].

Since T-cell mediated immunity provides critical protective immune response against *M*. *tuberculosis* infection [21, 22], the prime goal of TB vaccine development is to elicit effective long-lasting memory T-cells which could respond quickly and strongly upon encounter with *M*. *tuberculosis* [22, 23]. It has been reported that low dose of antigen favors the induction of central memory T cells (T_CM_) while high dose of antigen tents to stimulate effector memory T cells (T_EM_) or effective T cells formation [14, 24–26]. BCG vaccine mainly induces T_EM_ that survive for a shorter period of time than T_CM_ [5], which may underlie the limited duration of BCG vaccine protection [6]. In this study, we found that 24 weeks following immunization, the 2 μg LT69 vaccinated mice generated significantly higher levels of IFN-γ than the mice received 10 μg and 50 μg of the vaccine (*p* < 0.01), whereas the difference was not observed 6 weeks post immunization. Furthermore, thirty weeks post vaccination, low-dose LT69 (2 μg/dose) vaccination resulted in a 1.15 ± 0.09 log10 CFU reduction in lungs compared with PBS control, better than high-dose LT69 (0.85 ± 0.03 log10 CFU reduction) and BCG vaccine (0.53 ± 0.06 log10 CFU reduction). However, the differences in spleens among the groups of low-dose LT69 (1.01 ± 0.02 log10 CFU reduction), high-dose LT69 (0.78 ± 0.22 log10 CFU reduction) and BCG vaccine (0.89 ± 0.12 log10 CFU reduction) were not obvious. The reason might be that there are different memory T cell populations induced in different tissues following vaccines immunization. In addition, we challenged mice through respiratory tract, the bacteria load in lungs represents the protective effect against the aerosol attack, while the bacteria load in spleens might represent the protective effect against bacteria transmitted to spleen tissues through blood. These results suggested that low-dose of LT69 immunization generated more long-term T cell responses and consequently induced better protective efficacy than high-dose of LT69 and BCG vaccine in lung. In consistent with this，relative low dosage of other TB vaccine or vaccine candidate, such as BCG [27] and H56 [28], were reported to induce more stable, protective immunity than high dosages. The reason might be that low dosages of vaccine induced development of T_CM_ that led to long-term and high protection [14]. A recent study also reported that the recombinant BCG Δ*ureC*::*hly* vaccine-induced T_CM_ played the critical role on its long-term protection against pulmonary TB [6]. The dose related protective effect and mechanism were also verified in infection of other pathogens, such as influenza virus [24], simian immunodeficiency virus (SIV) [29], and *Leishmania major* [30].

In summary, multistage subunit vaccine LT69 consisting five antigens had strong immunogenicity and high protective efficacy against *M*. *tuberculosis*. Low-dose of LT69 generated long-term immune memory responses and showed higher protective efficacy against *M*. *tuberculosis* infection than BCG did at 30 weeks post vaccination. These findings have implications for design of new effective TB vaccines and vaccination regimens in the future.
